# Interaction between *BDNF* Polymorphism and Physical Activity on Inhibitory Performance in the Elderly without Cognitive Impairment

**DOI:** 10.3389/fnhum.2017.00541

**Published:** 2017-11-07

**Authors:** Anne Canivet, Cédric T. Albinet, Montserrat Rodríguez-Ballesteros, Christian Chicherio, Delphine Fagot, Nathalie André, Michel Audiffren

**Affiliations:** ^1^Université de Poitiers, Centre de Recherches sur la Cognition et l’Apprentissage, CNRS UMR 7295, Poitiers, France; ^2^Laboratoire Sciences de la Cognition, Technologie, Ergonomie (SCoTE), Université de Toulouse, INU Champollion, Albi, France; ^3^Laboratoire CiMoTheMA – EA 3808, CHU de Poitiers, Université de Poitiers, Groupe Génétique des Maladies Rares, Poitiers, France; ^4^Neuropsychology Unit, Neurology Clinic, Department of Clinical Neurosciences, Geneva University Hospitals, Geneva, Switzerland; ^5^Center for Interdisciplinary Study of Gerontology and Vulnerability, University of Geneva, Geneva, Switzerland; ^6^Maison des Sciences de l’Homme et de la Société, CNRS USR 3565, Université de Poitiers, Poitiers, France

**Keywords:** *BDNF* gene, physical activity, aging, executive functions, controlled inhibition, genetic polymorphism, reaction time

## Abstract

**Background:** In the elderly, physical activity (PA) enhances cognitive performances, increases brain plasticity and improves brain health. The neurotrophic hypothesis is that the release of brain-derived neurotrophic factor (BDNF), which is implicated in brain plasticity and cognition, is triggered by PA because motoneurons secrete BDNF into the bloodstream during exercise. Individual differences in cognitive performance may be explained by individual differences in genetic predisposition. A single nucleotide polymorphism on the *BDNF* gene, *BDNF*Val66Met, affects activity-dependent BDNF secretion. This study investigated the influence of the *BDNFVal66Met* polymorphism on the relationship between PA and controlled inhibition performance in older adults.

**Methods:** A total of 114 healthy elderly volunteers (mean age = 71.53 years old) were evaluated. Participants were genotyped for the *BDNFVal66Met* polymorphism. We evaluated inhibitory performance using choice reaction times (RT) and error rates from a Simon-like task and estimated their PA using two self-reported questionnaires. We established four groups according to PA level (active vs. inactive) and *BDNFVal66Met* genotype (Met carriers vs. Val-homozygous). The results were analyzed using ANOVA and ANCOVA, including age, gender and body mass index as covariates.

**Results:** The *BDNFVal66Met* polymorphism interacted with PA on controlled inhibition performance. More specifically, inactive Val-homozygous participants exhibited a lower inhibition performance than active Val homozygotes and inactive Met carriers; the former had a higher error rate without differences in RT.

**Conclusion:** Differences between individuals on inhibitory performance may be partially understood by the interaction between genetic influence in BDNF secretion and PA level. The results of this study clearly support the neurotrophic hypothesis that BDNF synthesis is an important mechanism underlying the influence of physical activity on brain structure and functions.

## Introduction

Substantial research demonstrated that regular physical activity (PA) exerts beneficial effects on cognitive performance in the elderly (Kirk-Sanchez and McGough, 2014; Erickson et al., 2015; Prakash et al., 2015). More specifically, it has been shown that PA exerts a larger positive influence on tasks mainly tapping executive functions than other tasks focusing on speed of information processing or visuospatial processing (Colcombe and Kramer, 2003). Different authors suggested that executive functions are not a unitary function but rather an umbrella of inter-correlated functions such as controlled inhibition, cognitive flexibility and updating of working memory (Duncan et al., 1997; Miyake et al., 2000). Within this diversity of executive functions, controlled inhibition seems to benefit more selectively from PA than the other executive functions (Smiley-Oyen et al., 2008; Boucard et al., 2012; Predovan et al., 2012; Gajewski and Falkenstein, 2016). Controlled inhibition is the capacity to suppress irrelevant information or prepotent responses, and it plays a core role in the executive functions construct (Miyake et al., 2000). Deterioration in inhibitory processes may play a central role in age-related declines in several different cognitive functions (Hasher and Zacks, 1988). However, some recent meta-analyses failed to demonstrate consistent and reliable benefits of PA on executive functions (Angevaren et al., 2008; Kelly et al., 2014), despite the apparent consensus of the positive effects of PA on cognitive functioning in elderly populations. Differences in study designs, training programs or the methodology used to analyze the principal outcomes primarily explain these inconsistent results (Audiffren et al., 2011). Another way explaining these discrepancies is the variety of participant characteristics in these studies, such as gender and age (see Fagot et al., 2017), or education level. The genetic profile is also an important factor that influences cognition, but it has received very little attention.

Several brain molecular and cellular mechanisms, such as the synthesis of neurotrophic proteins, progressively change during the aging process (Grady, 2008). Brain-derived neurotrophic factor (BDNF) is one of the main proteins involved in cerebral functioning (Neeper et al., 1995; Adlard et al., 2005; Snigdha et al., 2014), and its secretion decreases during aging (Pang and Lu, 2004). BDNF release is associated with the genetic profile of its gene. In other respect, regular PA was also associated with higher concentration of BDNF in the brain and enhancement of brain plasticity (Cotman and Berchtold, 2002; Cotman et al., 2007).

A single nucleotide polymorphism in the *BDNF* gene, *BDNFVal66Met*, affects BDNF secretion, and it may be involved in the deleterious effect of brain aging (Miyajima et al., 2008). The *Met* allele of the *BDNFVal66Met* polymorphism is present on one or two gene copies in 30% of the United States population (Shimizu et al., 2004). The deleterious effect of this *Met* allele reduces BDNF secretion in response to neuronal stimulation in adults (Egan et al., 2003; Kleim et al., 2006). When brain BDNF levels decrease, a lower cognitive performance can thus be expected. As mentioned just earlier, a lower level of brain BDNF can be observed in sedentary people by comparison to active people (Szuhany et al., 2015; Dinoff et al., 2016) and/or in people with two Met alleles at codon 66 of the BDNF gene. The examination of a possible interaction between PA and BDNF polymorphism on different cognitive functions declining with aging, and more particularly executive functions, is then particularly important. For instance, it can be expected that the positive effect of PA on executive functions may counteract the negative effect of the BDNF polymorphism Met allele (Raz et al., 2009). In this perspective, several authors examined the interaction between *BDNF*Val66Met and PA in elderly participants on cognitive performance, brain volume and/or cerebral plasticity (Kim et al., 2011; Erickson et al., 2013; Brown et al., 2014; Canivet et al., 2015; Crispim Nascimento et al., 2015; Thibeau et al., 2016). Only the last study of Thibeau et al. (2016) tested this interaction on executive functions. These authors demonstrated in a longitudinal study that *Val* carriers without PA exhibited poorer executive functions performance at age 75 than their peers with higher PA levels. In contrast, the level of PA did not affect executive functions performance in *Met-*heterozygous. It is interesting to note that they used four tasks tapping executive functions including two tasks well-known to involve controlled inhibition, the Stroop task and the Hayling sentence completion test. The results observed by Thibeau et al. (2016) converge with those of a previous study conducted by our group (Canivet et al., 2015), which demonstrated that only *Val-*homozygous older adults benefited from regular PA and exhibited better episodic memory performance compared to their *Val*-homozygous inactive counterparts. However, a same polymorphism can lead to notable variations in protein secretion in different ethnic groups (see Tsai et al., 2008). Consequently, a replication of Thibeau et al. (2016) data in a different population is important to generalize the results.

The present study used the same pool of participants aged 55 years and older than the study of Canivet et al. (2015) and examined the interaction between PA and the *BDNF* polymorphism on controlled inhibition performance using a modified Simon-like reaction time task. Participants in Simon-like tasks respond to a relevant stimulus feature (e.g., an arrow), and the position of the stimulus is always irrelevant (Verbruggen et al., 2005). There is a dimensional overlap between the irrelevant stimulus feature, i.e., the position of the stimulus, and the response set, i.e., the direction of the arrow in the example. When the stimulus position corresponds with the response side (e.g., left key press to a stimulus that is presented on the left of the screen), the trial is considered congruent, and the responses are typically faster than incongruent trials, where there is a mismatch between the stimulus position and response side. The dual-route model (e.g., Kornblum et al., 1990) suggests that the relevant stimulus feature (e.g., the direction of the arrow) is processed via a controlled route and activates the correct response, and a second response code is activated by the position of the stimulus via an automatic route. Ridderinkhof (2002) proposed that the automatic activation of the incorrect response is selectively suppressed by a central active inhibitory mechanism that requires time and may explain why participants respond slower on incongruent trials. The performance of older adults is consistently impaired in this type of task, but PA potentially reduces this impairment. It is also important to note that there is a problem of task impurity related to the measurement of the effectiveness and/or efficiency of a specific cognitive process (Burgess, 1997; Phillips, 1997). Generally, a cognitive task well-known to tap a cognitive process such as executive functions, or more specifically controlled inhibition, also involves other non-executive cognitive processes, for instance semantic memory. Consequently, a low performance observed in a specific task can be due to the executive component of the task and/or to the non-executive component of the task. The impurity of cognitive tasks may explain, on the one hand, the weak correlation between tasks well-known to tap executive functions (Miyake and Shah, 1999) and, on the other hand, the weak reliability test–retest of these same tasks (Denckla, 1996). It is recommended to use several tasks tapping the same cognitive function in the same study in order to avoid this problem of impurity. Another approach is to replicate the results using one or more tasks tapping the same cognitive process than the replicated study; this was the approach chosen in the present study.

The results of Canivet et al. (2015) and Thibeau et al. (2016) suggest a PA × *BDNF* polymorphism interaction on cognitive performance such as the effect of PA on controlled inhibition will be significant only for the *Val-*homozygous carriers, and Met carriers will not be affected by PA. In this perspective, the present cross-sectional study aims to replicate the results obtained by Thibeau et al. (2016) on controlled inhibition but using a different task tapping the controlled inhibition function in a sample of French participants.

## Materials and Methods

### Study

Data were collected within the “PRAUSE” survey conducted in Poitou-Charentes, France from 2011 to 2013 (Canivet et al., 2015). A total of 466 retired volunteers aged 55 years and older (mean age = 75.72; *SD* = 9.84) were included in the survey. They have been contacted through a sampling frame of 3716 address records from the census surveys conducted by the French Institute of Statistics and Economical Studies (Institut national de la statistique et des études économiques – INSEE). This master-sample for regional extensions was very representative of the senior population of Poitou-Charentes (France). The inclusion criteria of the study were: (1) to be 55 years old or more, (2) to live in Poitou-Charentes (France), (3) to be retired, in a situation of long-term unemployment, long-term sick leave or never engaged in the paid labor force. Non-native French speakers and people institutionalized in an Establishment of Accommodation for Dependent Old Persons (EHPAD) were excluded from participation. The survey was conducted at home and involved three sessions of 1.5–2 h each. A battery of cognitive tests and questionnaires was administered during these three sessions. Buccal swabs were taken during session 1 and the reaction time task was administered in session 3. There was an attrition rate of 39% between sessions 1 and 2, and 30% between sessions 2 and 3.

#### Participants

**FIGURE 1:**
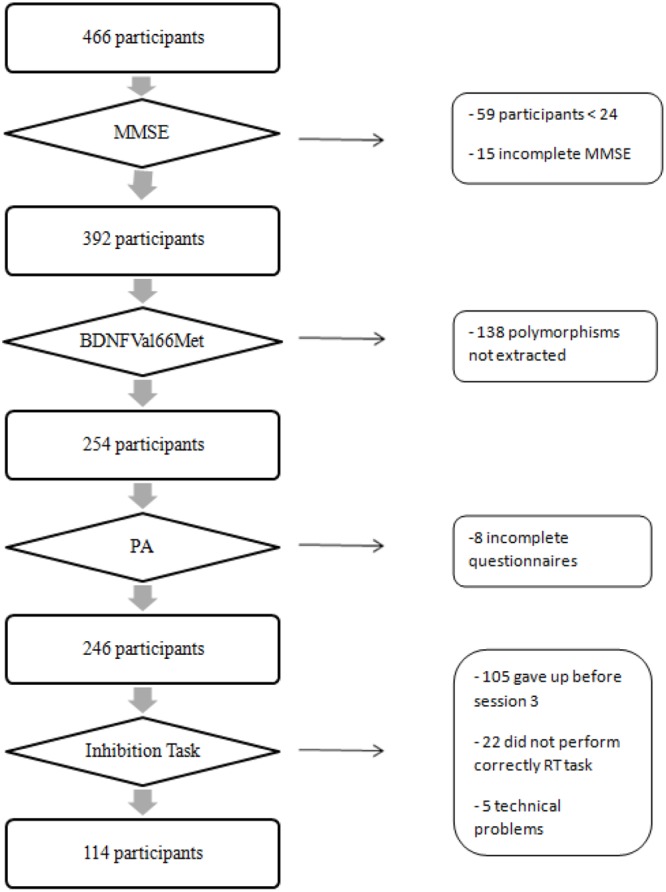
Flow chart describing the selection process of participants. MMSE, mini mental state examination; *BDNF*Val66Met, brain-derived neurotrophic factor gene polymorphism; PA, level of physical activity.

A total of 114 participants (mean age = 71.53 years; *SD* = 9.13) of the 466 volunteers were included in the current analyses because they completed all required tests and questionnaires to test our hypothesis (see **Figure 1**). We selected only active participants with body mass index (BMI) less than 35 to increase the likelihood of observing a significant effect of PA on cognitive performance (Nguyen et al., 2014).

This study was carried out in accordance with the recommendations of “Conseil National de l’Information Statistique” (CNIS) [French National Council of Statistical Information] and “Commission Nationale de l’Informatique et des Libertés” (CNIL) [French National Commission on Informatics and Liberty] with written informed consent from all subjects. All subjects gave written informed consent in accordance with the Declaration of Helsinki. The protocol was approved by two national ethics committees: (1) the survey received the “general interest and statistical quality” label from the CNIS (Visa n°2012X907RG); and (2) authorization n°1593815 from the CNIS (deliberation n°2012-375).

### Cognitive Assessments

#### Mini Mental State Examination

Global cognitive functioning was evaluated during the first session using the MMSE. Cognitive impairment (exclusion criterion) was defined as a score below 24 (Folstein et al., 1975). Only participants with an MMSE score equal to or higher than 24 were included in the study.

#### Controlled Inhibition

**FIGURE 2:**
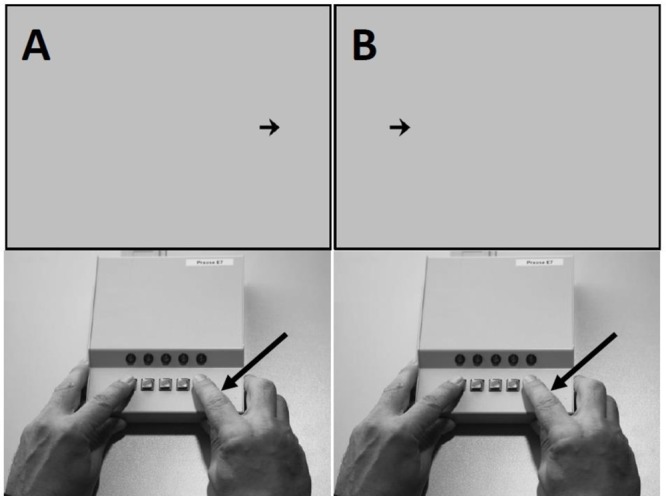
Setup of the reaction time task. **(A)** The direction of the arrow (toward the right side of the screen) is congruent with the location of the arrow (in the right side of the screen); the participant responds to the arrow by pressing the right key. **(B)** The direction of the arrow (toward the right side of the screen) in incongruent with the location of the arrow (in the left side of the screen); the participant responds to the arrow by pressing the right key. Congruent and incongruent stimuli had the same probability of occurrence in the right and the left side of the screen.

Inhibitory performance was assessed using a computerized reaction time task. The task was constructed and administered using E-Prime 2.0 software (Psychology Software Tools, Pittsburgh, PA, United States). The stimuli were arrows that appeared on the right or left of the computer screen. Participants responded as accurately and as rapidly as possible using a button press with a millisecond precision (Serial Response Box^TM^, Psychology software Tools, Pittsburgh, PA, United States) according to the direction indicated by the head of the arrow. If the arrow pointed to the right, the participant had to press the right key of the Serial Response Box with the right forefinger and if the arrow pointed to the left, he/she had to press the left key with the left forefinger (see **Figure 2**). On each trials, the following sequence of events occurred: a black fixation point appeared at the center of the screen for a mean duration of 500 ms, varying randomly between 300 and 700 ms with a 100 ms incremental step. The stimulus (arrow pointing to the left or right) appeared on the right or left side of the screen and remained until the onset of the participant’s response (see **Figure 2**). Afterward, the screen went blank for 1000 ms following the onset of the participant’ response or after a maximum of 2500 ms. There was a distance of approximatively 750 mm between the screen and the eyes of the participant. The stimulus (the arrow) was 20 mm wide and located at 137 mm from the center of the monitor. Measures of inhibition were obtained in one block of 300 trials, where 200 congruent trials and 100 incongruent trials were randomly presented. Congruent trials were trials where the arrow pointed to the right (→) and appeared on the right of the screen or pointed to the left (←) when appearing on the left of the screen. Incongruent trials were trials where the arrow pointed to the opposite side of its spatial location on the screen. We used mean reaction time (RT, in ms) data and error rate (in %) for incongruent and congruent trials as dependent variables. **Figure 2** described the experimental setup of the reaction time task.

### Genotyping

DNA was extracted from buccal cells using a QIAamp DNA Blood Mini Kit (QIAGEN GmbH - QIAGEN Strasse 1. 40724 Hilden, Germany) according to the manufacturer’s protocol. SNPs were genotyped using polymerase chain reaction and restriction fragment length polymorphism (PCR-RFLP) analysis. PCR amplification of the *BDNF* polymorphism *Val66Met* (rs6265) was performed using the forward primer 5′-GCCTACCCAGGTGTGCGG-3′ and the reverse fluorescent primer 5′-FAM-GAGGAGGCTCCAAAGGCAC-3′. PCR products were digested using the restriction enzyme Hsp92II (Promega Corporation – 2800 Woods Hollow Road. Madison, WI 537 11-5399, United States) and resolved using capillary electrophoresis in an ABI PRISM 3130 DNA Genetic Analyzer (Applied Biosystem by Life Technologie, Thermo Fisher Scientific – 168 Third Avenue, Waltham, MA, United States 02451).

The allelic frequency was estimated using Hardy Weinberg equilibrium and the khi^2^ test. The distribution of genotypes in the sample did not differ from the Hardy–Weinberg equilibrium (*p* = 0.65). Genetic data were analyzed using a dominance model that combined *Met* carriers into a single group (*Met/Met* + *Val/Met*) because the *Val/Met* and *Met/Met* genotypes were previously associated with decreased cognitive performance compared to *Val-*homozygous genotypes (Miyajima et al., 2008; Raz et al., 2009; Lim et al., 2014) and the low presence of the *Met/Met* genotype (Shimizu et al., 2004) in the population.

### Physical Activity

#### The Historical Leisure Activity Questionnaire (HLAQ)

The level of current PA was evaluated during the second session using the HLAQ (Kriska et al., 1988). This validated questionnaire was used to assess the history of PA weighted by their relative intensity. Participants were asked to report the frequency, type, intensity, and hours of PA performed during the present year. We used the Compendium of Physical Activities Tracking Guide 2011 (Ainsworth et al., 2011) to obtain a specific metabolic equivalent (MET) for each PA. We calculated the average energy expenditure (Mets-h/week) for each participant using the HLAQ data and the compendium. We classified the participants according to their METs-h/week following World Health Organization (WHO) recommendations: participants with greater than or equal to 7.5 METs-h/week were in the active group and participants with lower than 7.5 METs-h/week were in the inactive group.

#### The NASA/JSC Physical Activity Scale

All participants were asked to rate their current regular weekly physical practices during the first session to identify their PA level on a score from 0 to 7 using the NASA/JSC Physical Activity Scale (Jackson et al., 1990). We used these data to confirm the participant’s PA level in cases they were near the 7.5 METs-h/week (±0.50). Seventy-eight participants were evaluated using this verification method. They were considered active if their NASA/JSC PA score was strictly higher than 3. Participants who scored level 4 or above practiced regularly intensive PA at least 3 h per week (three participants). Participants were classified as inactive if their NASA/JSC PA score was strictly lower than 3 (one participant).

### Group Constituents

We established four independent groups of participants according to their PA level (above or below 7.5 METs-h/week) and *BDNFVal66Met* profile (*Met* Carriers vs. *Val-*homozygous) (see **Table 1**).

**Table 1:** Demographic characteristics of participants.

	Active		Inactive		Total	Effects of PA and BDNF
Groups	Met carriers	Val/Val	Met carriers	Val/Val		
Participants (n)	34	30	22	28	114	
Age (years)	70,85 (8.48)	68.98 (8.95)	71.83 (9.19)	74.84 (9.47)	71.53 (9.13)	PA^∗^
Gender (M/F)	22/13	15/15	11/11	8/20	56/58	PA^∗^, BDNF^∗^
Visual acuity (N/W)	27/7	25/5	16/6	22/6	90/24	*NS*
BMI (kg/m^2^)	26.01 (3.73)	27.23 (4.37)	28.94 (4.17)	28.92 (4.67)	27.57 (4.35)	PA^∗^
MMSE (0–30)	28.06 (1.58)	28.40 (1.48)	28.59 (1.18)	27.71 (2.05)	28.17 (1.63)	*NS*
Depression (0–30)	5.82 (4.85)	6.93 (5.40)	7.59 (5.40)	7.32 (4.39)	6.82 (4.98)	*NS*
Education level (1–20)	10.09 (3.52)	11.60 (3.64)	11.18 (4.02)	10.14 (3.61)	10.71 (3.69)	*NS*
Hour/week of PA	9.35 (5.59)	11.97 (8.76)	0.17 (0.42)	0.55 (0.70)	6.16 (7.48)	PA^∗^
Mets-h/week of PA	48.53 (31.02)	62.20 (50.28)	0.61 (1.56)	2.24 (2.70)	31.78 (40.91)	PA^∗^

*Mean (± standard deviation); M, male; F, female; N, normal vision with or without correction; W, weakened vision even with correction; BMI, Body Mass Index; PA^∗^, significant main effect of PA; BDNF^∗^, significant main effect of BDNF polymorphism; NS, no effect of PA or BDNF.*

### Geriatric Depression Scale (GDS)

The French version of the GDS (Bourque et al., 1990) was used in order to assess the level of participants’ depressive symptomatology (maximum = 30; Yesavage et al., 1983). Depression was evaluated because previous studies demonstrated an interaction between BDNF polymorphism and PA on depression (Bryan et al., 2007; Mata et al., 2010; Erickson et al., 2012). Brink et al. (1982) suggested that depression scores from 1 to 10 be considered normal, while depression score ≥ 11 are indicative of possible depression.

### Education Level

Education level contributes to cognitive reserves (Farfel et al., 2013; Stern, 2013), and it strongly influences cognitive performance in older adults. Education level was evaluated as the number of years of formal education from the 1st year of elementary school (level 1) to the 3rd year of a Ph.D. degree (level 20) (1–20 years).

### Statistical Analysis

The assumption of data normality and homogeneity was assessed using Lilliefors and Levene tests, respectively. Error rates were not normally distributed, and these data were arcsine square-root transformed. We performed separate analyses of variance (ANOVAs) on mean RT and transformed error rates using a general linear model, with PA level (active vs. inactive) and *BDNF* polymorphism (*Met* carriers vs. *Val-*homozygous) as between-subjects factors and trial type (congruent vs. incongruent) as a within-subject factor. Analyses of covariance (ANCOVAs) were performed on the same measures, with gender, age and BMI as covariates because these factors were significantly associated with PA or *BDNF* polymorphism (see **Table 1**). The level of significance was set at *p* < 0.05, and partial estimated effect sizes ($\eta_{p}^{2}$) are reported for significant results. *Post hoc* mean comparisons were performed using Bonferroni corrections for multiple comparisons. All statistical and power analyses were conducted with Statistica 7.1 package (Statsoft France, 2016).

## Results

The ANOVA on RT data revealed no main effect of PA [*F*(1,110) = 2.23, *p* > 0.13] or *BDNF* polymorphism [*F*(1,110) = 0.46, *p* > 0.50]. There was a significant effect of trial type. Congruent trials produced shorter RTs (*M* = 614.97 ms; *SD* = 11.60 ms) than incongruent trials (*M* = 770.26 ms; *SD* = 16.41 ms) [*F*(1,110) = 314.45; *p* < 0.05; $\eta_{p}^{2}$ = 0.77]. The PA × *BDNF* polymorphism × trial type interaction was marginally significant [*F*(1,110) = 3.184, *p* = 0.08], which suggests that only *Val-*homozygous active participants exhibited shorter RT on incongruent trials compared to their *Val-*homozygous inactive counterparts. However, the results of the ANCOVA revealed that this interaction was not significant when controlling for age, gender and BMI [*F*(1,102) = 1.88, *p* > 0.18]. A last ANCOVA on RT data with Visual acuity and Depression as additional covariates, pointed out no significant effect of Visual acuity [*F*(1,100) = 0.30; *p* > 0.58] and a marginal effect of Depression [*F*(1,100) = 3.51; *p* = 0.06] on RT (**Table 2**).

**Table 2:** Mean RT (± standard deviation) for congruent and incongruent trials as a function of group of participants.

	RT congruent trials	RT incongruent trials
Active Met carriers	601.27 (104.10)	757.99 (172.32)
Inactive Met carriers	610.82 (144.43)	756.48 (181.19)
Active Val/Val	594.48 (109.76)	728.29 (155.03)
Inactive Val/Val	649.04 (138.15)	828.59 (184.18)

*Mean (± standard deviation); RT, reaction time (in ms).*

**FIGURE 3:**
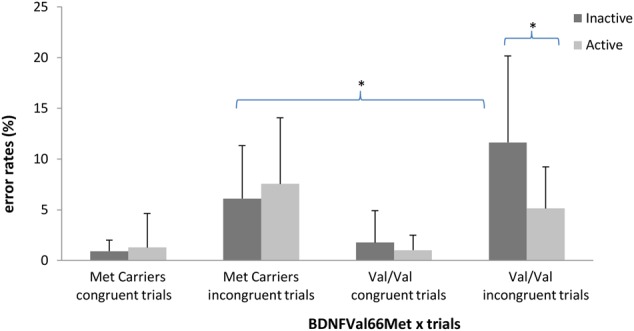
Interaction between *BDNF* polymorphism (Met carriers vs. Val/Val) and Physical Activity (active vs. inactive) on average error rate (in %) in congruent and incongruent trials. Errors bars represent standard deviation. ^∗^*p* < 0.01.

The ANOVA on error rates revealed no main effect of PA [*F*(1,110) = 2.90, *p* > 0.09] or *BDNF* polymorphism [*F*(1,110) = 1.49, *p* > 0.22]. The effect of trial type was significant [*F*(1,110) = 337.34; *p* < 0.05; $\eta_{p}^{2}$ = 0.75]. Participants made fewer errors for congruent trials (*M* = 1.24%; *SD* = 0.24%) than incongruent trials (*M* = 7.61%; *SD* = 0.60%). The PA × *BDNF* polymorphism × trial type interaction reached significance [*F*(1,110) = 9.75, *p* < 0.05, $\eta_{p}^{2}$ = 0.08]. The results of the ANCOVA revealed that this interaction remained significant after controlling for age, gender and BMI [*F*(1,102) = 6.72, *p* < 0.05, $\eta_{p}^{2}$ = 0.06]. The statistical power of this interaction was 72.8%. *Post hoc* analyses confirmed a significant difference in error rate between *Val/Val* active and *Val/Val* inactive participants only for the incongruent trials (*p* < 0.01) and a significant difference between *Val/Val* inactive participants and *Met* carrier inactive participants only for the incongruent trials (*p* < 0.01) (see **Figure 3**). A last ANCOVA on error rates with Visual acuity and Depression as additional covariates, pointed out no significant effect of Visual acuity [*F*(1,100) = 0.25; *p* > 0.61] and a significant effect of Depression [*F*(1,100) = 4.03; *p* < 0.05; $\eta_{p}^{2}$ = 0.05]. The interaction between PA × *BDNF* polymorphism × trial type remained significant despite controlling for these two additional covariates [*F*(1,100) = 6.70; *p* < 0.05; $\eta_{p}^{2}$ = 0.06].

## Discussion

This study examined the interaction between PA and the *BDNFVal66Met* polymorphism on controlled inhibitory performance in older adults. Our hypothesis was that PA would interact with the *Val* allele on cognitive performance that involved conflict monitoring and controlled inhibition (i.e., the incongruent trials in our experimental task). The classical detrimental effect of no-dimensional overlap between the direction and location of the stimuli (i.e., an arrow) was clearly reproduced and demonstrated using RT and error rates. The dual-route model states that incongruent trials require more time to inhibit an automatic response, which leads to slower RT and a higher percentage of errors because of the failure of the inhibition process in some trials (Ridderinkhof, 2002). However, we did not observe a clear significant interaction between PA and polymorphisms on the average RT for trials that involved controlled inhibition, but the interaction reached significance on error rates. Specifically, the interaction demonstrated that inactivity was associated with a significant increase in error rate only for inactive *Val-*homozygous participants in the incongruent condition. This interaction confirms that PA modulates the relationship between the BDNF polymorphism and controlled inhibition as measured using error rates for incongruent trials. More precisely, it suggests that PA and BDNF share a common neurophysiological mechanism that influences controlled inhibition. The most plausible mechanism that could play this role is the neurotrophic influence of BDNF on prefrontal neurons. Both exercise and the Val allele of the BDNF polymorphism increase the concentration of BDNF in the brain (Cotman and Berchtold, 2002), consequently they must interact at a structural and/or a functional level in some regions of the brain, in this case prefrontal regions. Then, the interaction between PA and BDNF polymorphism observed in the present study gives a supplementary argument for the mediation role of BDNF in the causal link between exercise and cognition.

In addition, the present results confirm and extend the results obtained by Thibeau et al. (2016): inactive *Val-*homozygous exhibited a poorer performance in controlled inhibition than Met carriers and active Val homozygous. In the current study, the degraded performance corresponded to a higher error rate for incongruent trials in a Simon-like task. Thibeau et al. (2016) observed very similar results using a Stroop task and a Hayling sentence completion task. These two studies strongly suggest that *Val-*homozygous individuals are more sensitive to PA level. Inactivity seems very deleterious for *Val-*homozygous carriers. Our results should be interpreted in light of the fact that physical inactivity is a new phenomenon from an evolutionary point of view. In contrast, the human genome is a product of a long evolutionary process that has remained stable for 10.000 years (Ratey and Loehr, 2011) and was built for hunters with periods of intense PA balanced with periods of rest. Therefore, the wild type *Val/Val* individuals could suffer more strongly from the effects of a sedentary lifestyle than Met carriers.

But why Met carriers did not demonstrate any positive effect of PA or negative effect of inactivity? The Met allele of the *BDNF* polymorphism is generally viewed as a deleterious variant because the *Met* allele reduces BDNF secretion in response to neuronal stimulation (Egan et al., 2003; Kleim et al., 2006). It is important to note that this deleterious effect of the Met allele was not observed in the present experiment. However, it could be hypothesized that BDNF secretion level in *Met* carriers may not increase sufficiently in response to regular PA, and the BDNF rate would remain too low to benefit cognitive performance. Another mechanism may explain the similar performance of controlled inhibition in active and inactive *Met* carriers. Brown’s hypothesis (Brown et al., 2014) is that the mature BDNF form is altered and inefficiently binds to the BDNF receptor (TrkB) in BDNF *Met* carriers, but the precursor form of BDNF (proBDNF) remains unaltered in BDNF *Met* carriers. Unfortunately, proBDNF binds its receptor (p75), and this link induces apoptosis. Therefore, Met carriers with high levels of PA may undergo higher levels of apoptotic changes in the brain because of the increasing proBDNF levels. Increasing mature BDNF levels in response to increased PA will not be sufficient to improve cognition. Notably, the Caucasian population exhibits an approximately 70% distribution of *Val*-homozygous (Shimizu et al., 2004). Val-homozygous carriers in our population exhibited the greatest benefit from the effects of PA, and 30% of the population (the Met carriers) was less or not sensitive to the effects of PA on controlled inhibition performance. Therefore, the cognitive improvements reported by epidemiological and interventional studies in primarily Western countries may be the result of the high percentage of BDNF Val-homozygous allele carriers in this population.

The present study emphasizes the interaction between BDNF polymorphism and PA as a set of life habits that is recognized as beneficial for brain health. We demonstrated that PA interacted with *BDNFVal66Met* on controlled inhibitory performance. Therefore, it is imperative to control for PA level to examine the putative impact of the *BDNFVal66Met* polymorphism on cognition in elderly populations.

Five main limitations may be noted. First, this study was cross-sectional. The interpretation of these results as a causal relationship between PA and cognition is limited by the study design; cross-sectional study do not allow establishing a causal relationship because better cognitive functioning may promote an individual to engage in PA. Future studies should use a randomized-controlled trial approach to determine whether BDNF *Val-*homozygous carriers benefit from exercise training programs. Second, the measure of PA was based on the self-report of participants. These factors induce the possibility of a biased reporting from the subjective perception of participants about their PA level. However, the HLAQ questionnaire and the NASA/JSC Physical Activity Scale are widely recognized and well-validated instruments. Third, some haplogroups may present protective or compensative effects to certain alterations in protein secretion that is influenced by a gene polymorphism. Tsai et al. (2008) demonstrated that the *Met* allele protected Asian-type populations from the deleterious effects of cognitive aging. It could be important to conduct replication of the present study and Thibeau’s et al. (2016) study in aging people from other ethnic group such as Asian-type population. Fourth, only one gene related to executive control was examined in the present study. However, executive control and the association between physical fitness and cognition are likely to be underpinned by many genes. Several genes have been identified in addition to BDNF: DRD4, DAT1, MAOA, COMT, APOE, each with a relatively small effect (for a review, Goldberg and Weinberger, 2004). It could be interesting to examine the synergistic and antagonistic actions of these different genes on cognition, and focus on those that share a common mechanism that may explain the causal link between exercise and cognition. However, these multi-polymorphisms studies require very large sample of participants.

The present report showed a significant interaction between PA and a *BDNF* gene polymorphism on controlled inhibitory performance. These data demonstrated that the association between PA and controlled inhibition was modulated by a *BDNF* polymorphism. Inactivity appeared more deleterious for Val-homozygous than *Met* carriers. This result improves our understanding of the neurophysiological mechanisms underlying the positive effect of PA on cognitive decline and provides brain BDNF as a convincing modulating candidate in this relationship.

## Author Contributions

MA: Scientific coordinator of the study, design of the protocol, data collection, data treatment, writing of the article, statistical analyses. AC: Data collection, data treatment, writing of the article, statistical analyses. CA: Design of the protocol, data collection, data treatment, writing of the article. MR-B: Data collection, determination of genetic polymorphism, writing of the article. CC and DF: Design of the cognitive task, data treatment, writing of the article. NA: Design of the protocol, data treatment, writing of the article.

## Conflict of Interest Statement

The authors declare that the research was conducted in the absence of any commercial or financial relationships that could be construed as a potential conflict of interest.

## References

[B1] Adlard P. A. Perreau V. M. Pop V. Cotman C. W. (2005). Voluntary exercise decreases amyloid load in a transgenic model of Alzheimer’s disease. *J. Neurosci.* 25 4217–4221. 10.1523/JNEUROSCI.0496-05.2005 15858047 PMC6725122

[B2] Ainsworth B. E. Haskell W. L. Herrmann S. D. Meckes N. Bassett D. R. Jr. Tudor-Locke C. (2011). Compendium of physical activities: a second update of codes and MET values. *Med. Sci. Sport Exer.* 43 1575–1581. 10.1249/MSS.0b013e31821ece12 21681120

[B3] Angevaren M. Aufdemkampe G. Verhaar H. J. J. Aleman A. Vanhees L. (2008). Physical activity and enhanced fitness to improve cognitive function in older people without known cognitive impairment. *Cochrane Database Syst. Rev.* 3:CD005381. 10.1002/14651858.CD005381.pub2 18646126

[B4] Audiffren M. André N. Albinet C. (2011). Effets positifs de l’exercice physique chronique sur les fonctions cognitives des seniors: bilan et perspectives. *Rev. Neuropsychol. Neurosci. Cogn. Clin.* 3 207–225. 10.3917/rne.034.0207

[B5] Boucard G. K. Albinet C. T. Bugaiska A. Bouquet C. A. Clarys D. Audiffren M. (2012). Impact of physical activity on executive functions in aging: a selective effect on inhibition among old adults. *J. Sport Exerc. Psychol.* 34 808–827. 10.1123/jsep.34.6.808 23204360

[B6] Bourque P. Blanchard L. Vézina J. (1990). Étude psychométrique de l’Échelle de dépression gériatrique. *Can. J. Aging* 9 348–355. 10.1017/S0714980800007467

[B7] Brink T. L. Yesavage J. A. Lum O. Heersema P. Adey M. B. Rose T. L. (1982). Screening tests for geriatric depression. *Clin. Gerontol.* 1 37–44. 10.1300/J018v01n01_06 7183759

[B8] Brown B. M. Bourgeat P. Peiffer J. J. Burnham S. Laws S. M. Rainey-Smith S. R. (2014). Influence of BDNF Val66Met on the relationship between physical activity and brain volume. *Neurology* 83 1345–1352. 10.1212/WNL.0000000000000867 25186863

[B9] Bryan A. Hutchison K. E. Seals D. R. Allen D. L. (2007). A transdisciplinary model integrating genetic, physiological, and psychological correlates of voluntary exercise. *Health Psychol.* 26 30–39. 10.1037/0278-6133.26.1.30 17209695 PMC1896050

[B10] Burgess P. W. (1997). “Theory and methodology in executive function research,” in *Methodology of Frontal and Executive Function* ed. Rabbitt P. (Hove: Psychology Press) 81–116.

[B11] Canivet A. Albinet C. T. André N. Pylouster J. Rodríguez-Ballesteros M. Kitzis A. (2015). Effects of BDNF polymorphism and physical activity on episodic memory in the elderly: a cross sectional study. *Eur. Rev. Aging Phys. Act.* 12 15. 10.1186/s11556-015-0159-2 26865879 PMC4748321

[B12] Colcombe S. Kramer A. F. (2003). Fitness effects on the cognitive function of older adults: a meta-analytic study. *Psychol Sci* 14 125–130. 10.1111/1467-9280.t01-1-01430 12661673

[B13] Cotman C. W. Berchtold N. C. (2002). Exercise: a behavioral intervention to enhance brain health and plasticity. *Trends Neurosci.* 25 295–301. 10.1016/S0166-2236(02)02143-4 12086747

[B14] Cotman C. W. Berchtold N. C. Christie L. A. (2007). Exercise builds brain health: key roles of growth factor cascades and inflammation. *Trends Neurosci.* 30 464–472. 10.1016/j.tins.2007.06.011 17765329

[B15] Crispim Nascimento C. M. Pereira J. R. de Andrade L. P. Garuffi M. Ayan C. Kerr D. S. (2015). Physical exercise improves peripheral BDNF levels and cognitive functions in mild cognitive impairment elderly with different BDNF Val66Met genotypes. *J. Alzheimers. Dis.* 43 81–91. 10.3233/JAD-140576 25062900

[B16] Denckla M. B. (1996). “A theory and model of executive function: a neuropsychological perspective,” in *Attention, Memory and Executive Function* eds Lyon G. Krasnegor N. (Towson, MD: Paul Brooks).

[B17] Dinoff A. Herrmann N. Swardfager W. Liu C. S. Sherman C. Chan S. (2016). The effect of exercise training on resting concentrations of peripheral brain-derived neurotrophic factor (BDNF): a meta-analysis. *PLOS ONE* 11:e0163037. 10.1111/ejn.13603 27658238 PMC5033477

[B18] Duncan J. Johnson R. Swales M. Freer C. (1997). Frontal lobe deficits after head injury: unity and diversity of function. *Cogn. Neuropsychol.* 14 713–741.

[B19] Egan M. F. Kojima M. Callicott J. H. Goldberg T. E. Kolachana B. S. Bertolino A. (2003). The BDNF val66met polymorphism affects activity-dependent secretion of BDNF and human memory and hippocampal function. *Cell* 112 257–269. 10.1016/S0092-8674(03)00035-7 12553913

[B20] Erickson K. I. Banducci S. E. Weinstein A. M. Macdonald A. W. Ferrell R. E. Halder I. (2013). The brain-derived neurotrophic factor Val66Met polymorphism moderates an effect of physical activity on working memory performance. *Psychol. Sci.* 24 1770–1779. 10.1177/0956797613480367 23907543 PMC3947596

[B21] Erickson K. I. Hillman C. H. Kramer A. F. (2015). Physical activity, brain, and cognition. *Curr. Opin. Behav. Sci.* 4 27–32. 10.1016/j.cobeha.2015.01.005

[B22] Erickson K. I. Miller D. L. Roecklein K. A. (2012). The aging hippocampus: interactions between exercise, depression, and BDNF. *Neuroscientist* 18 82–97. 10.1177/1073858410397054 21531985 PMC3575139

[B23] Fagot D. Chicherio C. Albinet C. T. André N. Audiffren M. (2017). The impact of physical activity and sex differences on intraindividual variability in inhibitory performance in older adults. *Neuropsychol. Dev. Cogn. B Aging Neuropsychol. Cogn.* 10.1080/13825585.2017.1372357 [Epub ahead of print]. 28868969

[B24] Farfel J. M. Nitrini R. Suemoto C. K. Grinberg L. T. Ferretti R. E. L. Leite R. E. P. (2013). Very low levels of education and cognitive reserve: a clinicopathologic study. *Neurology* 81 650–657. 10.1212/WNL.0b013e3182a08f1b 23873971 PMC3775692

[B25] Folstein M. F. Folstein S. E. McHugh P. R. (1975). “Mini-mental state”: a practical method for grading the cognitive state of patients for the clinician. *J. Psychiatr. Res.* 12 189–198. 1202204 10.1016/0022-3956(75)90026-6

[B26] Gajewski P. D. Falkenstein M. (2016). Physical activity and neurocognitive functioning in aging-a condensed updated review. *Eur. Rev. Aging Phys. Act.* 13 1–7. 10.1186/s11556-016-0161-3 26865880 PMC4748322

[B27] Goldberg T. E. Weinberger D. R. (2004). Genes and the parsing of cognitive processes. *Trends Neurosci.* 8 325–335. 10.1016/j.tics.2004.05.011 15242692

[B28] Grady C. L. (2008). Cognitive neuroscience of aging. *Ann. N. Y. Acad. Sci.* 1124 127–144. 10.1196/annals.1440.009 18400928

[B29] Hasher L. Zacks R. T. (1988). Working memory, comprehension, and aging: a review and a new view. *Psychol. Learn. Motiv.* 22 193–225. 10.1016/S0079-7421(08)60041-9

[B30] Jackson A. S. Blair S. N. Mahar M. T. Wier L. T. Ross R. M. Stuteville J. E. (1990). Prediction of functional aerobic capacity without exercise testing. *Med. Sci. Sports Exerc.* 22 863–870. 2287267 10.1249/00005768-199012000-00021

[B31] Kelly M. E. Loughrey D. Lawlor B. A. Robertson I. H. Walsh C. Brennan S. (2014). The impact of exercise on the cognitive functioning of healthy older adults: a systematic review and meta-analysis. *Ageing Res. Rev.* 16 12–31. 10.1016/j.arr.2014.05.002 24862109

[B32] Kim J. M. Stewart R. Bae K. Y. Kim S. W. Yang S. J. Park K. H. (2011). Role of BDNF val66met polymorphism on the association between physical activity and incident dementia. *Neurobiol. Aging* 32 551. e5–e12. 10.1016/j.neurobiolaging.2010.01.018 20172629

[B33] Kirk-Sanchez N. J. McGough E. L. (2014). Physical exercise and cognitive performance in the elderly: current perspectives. *Clin. Interv. Aging* 9 51–62. 10.2147/CIA.S39506 24379659 PMC3872007

[B34] Kleim J. A. Chan S. Pringle E. Schallert K. Procaccio V. Jimenez R. (2006). BDNF val66met polymorphism is associated with modified experience-dependent plasticity in human motor cortex. *Nat. Neurosci.* 9 735–737. 10.1038/nn1699 16680163

[B35] Kornblum S. Hasbroucq T. Osman A. (1990). Dimensional overlap: cognitive basis for stimulus-response compatibility - a model and taxonomy. *Psychol. Rev.* 97 253–270. 10.1037/0033-295X.97.2.253 2186425

[B36] Kriska A. M. Sandler R. B. Cauley J. A. Laporte R. E. Hom D. L. Pambianco G. (1988). The assessment of historical physical activity and its relation to adult bone parameters. *Am. J. Epidemiol.* 127 1053–1063. 10.1093/oxfordjournals.aje.a114881 3358406

[B37] Lim Y. Y. Villemagne V. L. Laws S. M. Ames D. Pietrzak R. H. Ellis K. A. (2014). Effect of BDNF Val66Met on memory decline and hippocampal atrophy in prodromal Alzheimer’s disease: a preliminary study. *PLOS ONE* 9:e86498. 10.1371/journal.pone.0086498 24475133 PMC3903533

[B38] Mata J. Thompson R. J. Gotlib I. H. (2010). BDNF genotype moderates the relation between physical activity and depressive symptoms. *Health Psychol.* 29 130–133. 10.1037/a0017261 20230085 PMC2847262

[B39] Miyajima F. Ollier W. Mayes A. Jackson A. Thacker N. Rabbitt P. (2008). Brain-derived neurotrophic factor polymorphism Val66Met influences cognitive abilities in the elderly. *Genes Brain Behav.* 7 411–417. 10.1111/j.1601-183X.2007.00363.x 17973920

[B40] Miyake A. Friedman N. P. Emerson M. J. Witzki A. H. Howerter A. Wager T. D. (2000). The unity and diversity of executive functions and their contributions to complex “frontal lobe” tasks: a latent variable analysis. *Cogn. Psychol.* 41 49–100. 10.1006/cogp.1999.0734 10945922

[B41] Miyake A. Shah P. (1999). “Toward unified theories of working memory: emerging general consensus, unresolved theoretical issues, and future research directions,” in *Models of Working Memory: Mechanisms of Active Maintenance and Executive Control* eds Miyake A. Shah P. (New York, NY: Cambridge University Press) 442–481.

[B42] Neeper S. A. Gomezpinilla F. Choi J. Cotman C. (1995). Exercise and brain neurotrophins. *Nature* 373 109. 10.1038/373109a0 7816089

[B43] Nguyen J. C. D. Killcross A. S. Jenkins T. A. (2014). Obesity and cognitive decline: role of inflammation and vascular changes. *Front. Neurosci.* 8:375 10.3389/fnins.2014.00375 PMC4237034 25477778

[B44] Pang P. T. Lu B. (2004). Regulation of late-phase LTP and long-term memory in normal and aging hippocampus: role of secreted proteins tPA and BDNF. *Ageing Res. Rev.* 3 407–430. 10.1016/j.arr.2004.07.002 15541709

[B45] Phillips L. H. (1997). “Do “frontal tests” measure executive function? Issues of assessment and evidence from fluency tests,” in *Methodology of Frontal and Executive Function* ed. Rabbitt P. (Hove: Psychology Press) 191–213.

[B46] Prakash R. S. Voss M. W. Erickson K. I. Kramer A. F. (2015). Physical activity and cognitive vitality. *Annu. Rev. Psychol.* 66 769–797. 10.1146/annurev-psych-010814-015249 25251492

[B47] Predovan D. Fraser S. A. Renaud M. Bherer L. (2012). The effect of three months of aerobic training on stroop performance in older adults. *J. Aging Res.* 2012:269815. 10.1155/2012/269815 23304504 PMC3530182

[B48] Ratey J. J. Loehr J. E. (2011). The positive impact of physical activity on cognition during adulthood: a review of underlying mechanisms, evidence and recommendations. *Rev. Neurosci.* 22 171–185. 10.1515/rns.2011.017 21417955

[B49] Raz N. Rodrigue K. M. Kennedy K. M. Land S. (2009). Genetic and vascular modifiers of age-sensitive cognitive skills: effects of COMT, BDNF, ApoE, and hypertension. *Neuropsychology* 23 105–116. 10.1037/a0013487 19210038 PMC2729285

[B50] Ridderinkhof K. R. (2002). “Activation and suppression in conflict tasks: empirical clarification through distributional analysis,” in *Attention & Performance: Common Mechanisms in Perception and Action* Vol. 19 eds Prinz W. Hommel B. (Oxford: Oxford University Press) 494–519.

[B51] Shimizu E. Hashimoto K. Iyo M. (2004). Ethnic difference of the BDNF 196G/A (val66met) polymorphism frequencies: the possibility to explain ethnic mental traits. *Am. J. Med. Genet. B Neuropsychiatr. Genet.* 126 122–123. 10.1002/ajmg.b.20118 15048661

[B52] Smiley-Oyen A. L. Lowry K. A. Francois S. J. Kohut M. L. Ekkekakis P. (2008). Exercise, fitness, and neurocognitive function in older adults: the “selective improvement” and “cardiovascular fitness” hypotheses. *Ann. Behav. Med.* 36 280–291. 10.1007/s12160-008-9064-5 18825471 PMC2748860

[B53] Snigdha S. de Rivera C. Milgram N. W. Cotman C. W. (2014). Exercise enhances memory consolidation in the aging brain. *Front. Aging Neurosci.* 6:3 10.3389/fnagi.2014.00003 PMC3910002 24550824

[B54] Statsoft France (2016). *STATISTICA (Data Analysis Software), Version 7.1.* Available at: www.statsoft.fr

[B55] Stern Y. (2013). Cognitive reserve: implications for assessment and intervention. *Folia Phoniatr. Logop.* 65 49–54. 10.1159/000353443 23941972 PMC3970779

[B56] Szuhany K. L. Bugatti M. Otto M. W. (2015). A meta-analytic review of the effects of exercise on brain-derived neurotrophic factor. *J. Psychiatr. Res.* 60 56–64. 10.1016/j.jpsychires.2014.10.003 25455510 PMC4314337

[B57] Thibeau S. McFall G. P. Wiebe S. A. Anstey K. J. Dixon R. A. (2016). Genetic factors moderate everyday physical activity effects on executive functions in aging: evidence from the victoria longitudinal study. *Neuropsychology* 30 6–17. 10.1037/neu0000217 26710092 PMC4693634

[B58] Tsai S.-J. Gau Y.-T. A. Liu M.-E. Hsieh C.-H. Liou Y.-J. Hong C.-J. (2008). Association study of brain-derived neurotrophic factor and apolipoprotein E polymorphisms and cognitive function in aged males without dementia. *Neurosci. Lett.* 433 158–162. 10.1016/j.neulet.2007.12.057 18242855

[B59] Verbruggen F. Liefooghe B. Notebaert W. Vandierendonck A. (2005). Effects of stimulus-stimulus compatibility and stimulus–response compatibility on response inhibition. *Acta Psychol.* 120 307–326. 10.1016/j.actpsy.2005.05.003 15993830

[B60] Yesavage J. A. Brink T. L. Rose T. L. Lum O. Huang V. Adey M. (1983). Development and validation of a geriatric depression screening scale: a preliminary report. *J. Psychiatr. Res.* 17 37–49. 10.1016/0022-3956(82)90033-4 7183759

